# The relationship between albumin corrected anion gap levels and mortality in patients with asthma in the ICU

**DOI:** 10.1038/s41598-023-44182-8

**Published:** 2023-10-06

**Authors:** Shidong Wang, Hong Teng, Hongyan Han, Yunjie Xu

**Affiliations:** Department of Respiratory Medicine, Shaoxing Second Hospital, Shaoxing, China

**Keywords:** Medical research, Risk factors

## Abstract

Although previous studies have suggested that albumin-corrected anion gap (ACAG) may be a predictor of mortality in critically ill patients in intensive care unit (ICU), its utility in the context of asthma has not been definitively established. In this study, baseline data, albumin concentration, anion gap (AG) and 30-d mortality data were retrieved from the Medical Information Mart for Intensive Care IV database (MIMIC-IV) for patients with asthma in the intensive care unit. Receiver operating characteristic (ROC) curves were constructed to analyze the predictive ability of ACAG and AG. The risk of 30-day mortality among patients with ACAG and asthma was analyzed using a restricted cubic spline (RCS) plot. Decision curve analysis (DCA) was used to evaluate the clinical usefulness of ACAG as a prognostic factor for 30-day mortality. Subsequently, subgroup analysis was conducted to explore potential variations in the relationship between ACAG and 30-day mortality based on factors such as sex, age, whether the asthma was acute, and other co-morbidities. Our study reveals that ACAG is a significant independent predictor of 30-day mortality in asthmatic patients receiving intensive care. The area under the AUC curve for ACAG was found to be 0.703, which is higher than that of AG, indicating that ACAG has a better predictive ability for 30-day mortality in this population. Furthermore, higher levels of ACAG were found to be associated with increased risk of 30-day mortality in asthmatic patients. In addition, decision curve analysis (DCA) demonstrated that the net benefit of ACAG was greater than that of AG. These findings suggest that ACAG may be a valuable prognostic factor for predicting 30-day mortality in asthmatic patients in the ICU. Our study provides evidence that ACAG is associated with an increased risk of 30-d mortality and has better predictive value in patients with combined asthma who are admitted to the ICU than AG.

## Introduction

Asthma is a prevalent respiratory disease affecting over 300 million individuals worldwide^1^. Despite some recent advances in treatment, asthma control remains suboptimal, with almost half of adult patients experiencing acute attacks annually^2^. The United States, for example, reports an estimated 25,000–50,000 cases of asthma requiring intensive care unit (ICU) treatment annually^3^. Hospital mortality rates for asthma vary by region, with a mean rate of 0.8% reported in a recent epidemiological study in^4^. Therefore, early identification of patients at risk of mortality in the asthma population and prompt treatment are crucial for reducing death rates.

In recent years, several biological markers, including IgE, FeNO, eosinophils, cytokines and osteopontin, have been introduced to asthma management^5^. Despite their potential usefulness, these markers have limitations, such as the scarcity of indicators related to patient mortality or difficulties in clinical detection. Therefore, there is a pressing need to identify new markers that can better predict patient prognosis. Anion gap (AG) is a well-established measure for determining acid–base balance disorders and is widely used in clinical practice due to its ease of calculation. However, recent studies have suggested that high AG levels are associated with mortality in various diseases, such as sepsis and end-stage renal disease. For instance, Zhu et al.^6^ concluded that serum AG concentration is an independent risk factor for 30-day and 90-day mortality in patients with sepsis, while Yohei Arai et al.^7^ found that AG was associated with mortality in patients with end-stage renal disease. In addition to the above diseases, AG is closely related to asthma. Asthma can affect the normal gas exchange of the human body, cause hypoxemia, respiratory muscle fatigue, anaerobic metabolism occurs, cause metabolic acidosis, and AG in patients is often increased^8^. High AG levels, which represent hypoxia and tissue hypoperfusion, are more common in patients with worsening asthma, suggesting a poor prognosis^9^.

Although AG is closely related to asthma, the level of AG is greatly affected by the level of albumin^10^, every 1 g/dl decrease in albumin concentration, AG will decrease about 2.5 mmol/L, hypoproteinemia can make AG false negative results. In addition, the use of AG in such patients with abnormal albumin levels is limited because albumin levels are often reduced in patients with asthma^11^. In order to correct the level of albumin levels change on AG, albumin correction anion gap this concept was put forward in clinic. In the field of cardiovascular disease, such as in patients with myocardial infarction, ACAG has been shown to be associated with 30-day mortality^12^. Lambert et al.^13^ also suggested that the use of ACAG could improve the efficacy of diagnosing the risk of some diseases. However, the relationship between ACAG and 30-d mortality in asthmatic patients remains uncertain. Therefore, this retrospective study aimed to investigate the relationship between ACAG and 30-day mortality in asthmatic patients in the intensive care unit.

## Methods

### Database

The present study utilized data from the MIMIC-IV database v2.0 (https://mimic.mit.edu/), which contains information on 76,540 hospitalizations of critically ill patients admitted to Beth Israel Deaconess Medical Center in Boston between 2008 and 2019. The database includes various patient information such asdemographics, laboratory indicators, vital signs, disease name, treatment measures, and survival. With the development of medical big data, various public databases have been applied to clinical research. The MIMIC database has the advantages of large amount of data and good data quality, and more and more researchers use it to carry out research^14^.Access to the database was granted to one of the authors, Shidong Wang, after successful completion of an online course and examination (Certificate ID: 12114387).The database preserves anonymity of patients and therefore, informed consent was not necessary. We conducted this study in accordance with the Strengthening the Reporting of Observational Studies in Epidemiology (STROBE)^15^.

### Study population

In the present study, we employed strict inclusion criteria. First, we included patients who had been diagnosed with asthma during their hospital admission from 2008 to 2019, and the diagnostic criteria were based on the International Classification of Diseases, Ninth and Tenth Revisions (refer to Supplementary Table 1). Second, we limited the age range to individuals who were 18 years or older. Lastly, we only considered patients who had albumin and anion gap measurements available within 24 h of admission to the ICU. In cases where the same patient was admitted multiple times, we included only information from the initial admission and the first ICU stay. These inclusion criteria were established to ensure the accuracy and reliability of our study results.

### Data extraction

Relevant patient information was extracted from the MIMIC-IV database using Navicat Premium 15.0 software. The extracted data included patient demographics such as age and gender, vital signs such as oxygen saturation and respiratory rate, disease severity scores such as sequential organ failure assessment (SOFA) score, comorbidity index such as Charlson comorbidity index (CCI), laboratory indicators such as albumin, anion gap, white blood cells, red blood cells, platelets, red blood cell distribution width, blood urea nitrogen, and bicarbonate. If there were multiple measurements of an index within 24 h, the mean value was taken to ensure accuracy. ACAG was calculated using the formula^10^: ACAG (mmol/l) = AG (mmol/l) + [4.4 − albumin (g/dl)] * 2.5, to adjust for the influence of albumin levels. The primary outcome of interest in this study was 30-d mortality in patients diagnosed with asthma.

### Statistical analysis

Multiple interpolation was employed to address missing values. The normality of variables was assessed using the Kolmogorov–Smirnov test. Normally distributed variables were expressed as mean ± SD and analyzed using the independent samples *t*-test; while non-normally distributed variables were expressed as median (interquartile range) and compared using the Wilcoxon rank sum test. Categorical variables are expressed as percentages and numbers and compared using the chi-square test. We conducted binary logistic regression analysis to identify independent risk factors associated with 30-day mortality in asthma patients. ROC curves were plotted for both AG and ACAG, and DCA was performed to compare the predictive value and net benefit of AG and ACAG in predicting 30d mortality in asthma patients. To investigate the association between ACAG and the risk of 30d mortality in patients with asthma, restricted three times spline plots were used. Additionally, subgroup analyses were conducted to explore the relationship between ACAG in different populations of asthma patients. Data analysis was performed using SPSS software (v27.0; IBM), MedCalc statistical software (v20.218; MedCalc Software LTD.) and R Software (ver 4.2.1). The DCA adopts R software and “*rmda*” package. *P* < 0.05 was considered statistically significant.

## Results

### Baseline characteristics

In this study, a total of 1561 patients were included, of whom 1326 were classified into the survival group, while 235 were in the death group, resulting in a 30-d mortality rate of 15%. Notably, patients in the death group tended to be older with higher AG and ACAG levels, but lower albumin levels when compared to the survival group. Detailed information on the baseline characteristics of the study population can be found in Table 1.

**Table 1:** Demographic and clinical characteristics of the study population.

Characteristics	Survival (n = 1326)	Death (n = 235)	*P*-value
Age, year	60.00 [47.00, 72.00]	68.00 [56.00, 80.00]	< 0.001
Gender (male, %)	799 (60.26)	150 (63.83)	0.336
SpO2, %	97 [96, 98]	97 [95, 98]	0.013
RR	19 [17, 22]	21 [18, 24]	< 0.001
ALB, g/dL	3.42 [3.00, 3.90]	3.00 [2.50, 3.48]	< 0.001
AG, mmol/L	14.50 [13.00, 17.00]	16.50 [14.00, 20.00]	< 0.001
ACAG, mmol/L	17.25 [15.12, 19.38]	20.62 [17.00, 24.00]	< 0.001
WBC, 10^9^/L	10.50 [7.48, 14.52]	12.53 [7.93, 17.62]	< 0.001
RBC, 10^9^/L	3.78 [3.28, 4.24]	3.42 [2.90, 4.03]	< 0.001
Platelet, 10^9^/L	208.88 [149.50, 272.25]	168.00 [109.62, 242.75]	< 0.001
RDW, %	14.40 [13.45, 15.86]	15.75 [14.18, 17.92]	< 0.001
BUN, mmol/L	17.50 [11.50, 28.00]	28.00 [17.75, 49.50]	< 0.001
Glucose, mg/dL	130.25 [106.50, 168.00]	138.50 [108.25, 186.50]	0.052
Sodium, mmol/L	138.50 [136.00, 141.00]	137.50 [134.00, 141.25]	0.058
Potassium, mmol/L	4.05 [3.75, 4.45]	4.30 [3.88, 4.90]	< 0.001
Bicarbonate, mmol/L	23.00 [20.00, 26.00]	21.00 [17.98, 24.00]	< 0.001
SOFA score	4.00 [2.00, 7.00]	9.00 [5.50, 13.00]	< 0.001
CCI	5.00 [3.00, 7.00]	8.00 [6.00, 9.00]	< 0.001

RR, Respiratory rate; ALB, albumin; AG, anion gap; ACAG, albumin corrected anion gap; WBC, white blood cell; RBC, red blood cell; RDW, red cell distribution width; BUN, blood urea nitrogen; SOFA, sequential organ failure assessment; CCI, Charlson comorbidity index.

### Analysis of risk factors associated with 30d mortality in patients with asthma

The indicators listed in Table 1 underwent univariate logistic regression analysis, and those with a *p* < 0.05 were then included in the multifactor logistic regression analysis. Due to co-collinearity with ACAG, AG was excluded from the multifactor logistic regression analysis. The binary logistic regression analysis revealed that ACAG was an independent risk factor for 30-day mortality in patients with asthma (OR = 1.07, 95% CI 1.02–1.12, *p* = 0.011). The rest of the binary logistic regression analysis results are shown in Table 2.

**Table 2:** Binary logistic regression analysis of 30-d mortality in patients with asthma.

Variable	Univariable		Multivariable	
	OR (95% CI)	*P*-value	OR (95% CI)	*P*-value
Age	1.03 (1.02–1.04)	*p* < 0.001	1.02 (1.01–1.03)	*p* = 0.005
Gender (male)	0.86 (0.64–1.15)	*p* = 0.301		
SpO2	0.858 (0.81–0.91)	*p* < 0.001	0.875 (0.82–0.94)	*p* < 0.001
RR	1.10 (1.07–1.14)	*p* < 0.001	1.05 (1.01–1.09)	*p* = 0.026
ALB	0.37 (0.30–0.46)	*p* < 0.001	0.68 (0.51–0.90)	*p* = 0.006
ACAG	1.19 (1.15–1.22)	*p* < 0.001	1.07 (1.02–1.12)	*p* = 0.011
WBC	1.03 (1.01–1.04)	*p* < 0.001	1.01 (1.00–1.03)	*p* = 0.132
RBC	0.57 (0.47–0.70)	*p* < 0.001	0.99 (0.77–1.26)	*p* = 0.911
Platelet	1.00 (1.00–1.00)	*p* < 0.001	1.00 (1.00–1.00)	*p* = 0.765
RDW	1.23 (1.17–1.29)	*p* < 0.001	1.10 (1.04–1.18)	*p* = 0.002
BUN	1.02 (1.01–1.02)	*p* < 0.001	1.00 (0.99–1.00)	*p* = 0.278
Glucose	1.00 (1.00–1.00)	*p* = 0.025	1.00 (1.00–1.00)	*p* = 0.776
Sodium	1.00 (0.97–1.02)	*p* = 0.729		
Potassium	1.68 (1.39–2.03)	*p* < 0.001	1.10 (0.87–1.40)	*p* = 0.424
Bicarbonate	0.92 (0.89–0.94)	*p* < 0.001	1.00 (0.96–1.04)	*p* = 0.840
SOFA	1.25 (1.21–1.30)	*p* < 0.001	1.17 (1.12–1.23)	*p* < 0.001
CCI	1.26 (1.20–1.32)	*p* < 0.001	1.17 (1.09–1.25)	*p* < 0.001

RR, Respiratory rate; ALB, albumin; AG, anion gap; ACAG, albumin corrected anion gap; WBC, white blood cell; RBC, red blood cell; RDW, red cell distribution width; BUN, blood urea nitrogen; SOFA, sequential organ failure assessment; CCI, Charlson comorbidity index.

### Analysis of the predictive value of ACAG for 30d day mortality in patients with asthma

**Figure 1:**
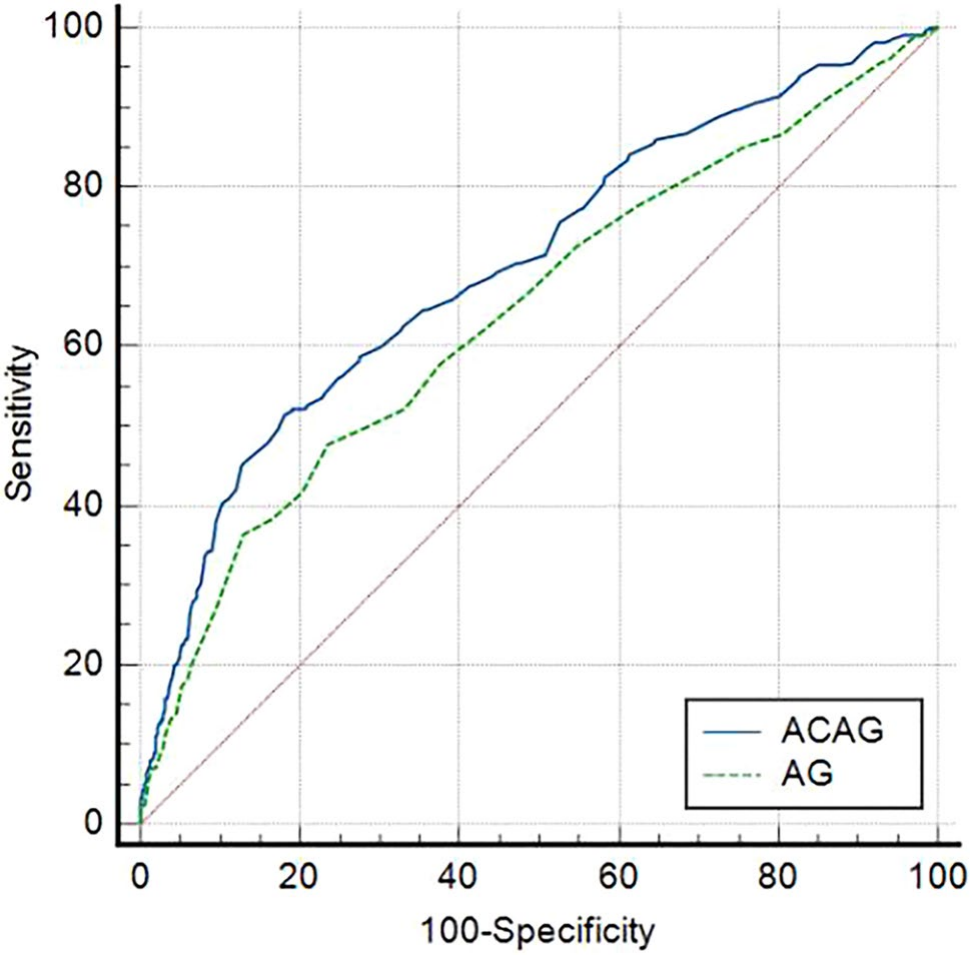
ROC curves for ACAG, AG.

In this study, the predictive value of ACAG for 30-day mortality in asthma patients was evaluated using ROC curves and compared with AG. The AUC areas for ACAG and AG were 0.703 and 0.642, respectively, indicating that ACAG had a better predictive value for 30-day mortality in asthma patients than AG (Z = 6.341, *P* < 0.001). The sensitivity of ACAG was 0.515 and the specificity was 0.819, respectively, both higher than those of AG. The optimal cut-off value for ACAG was 20.38 mmol/L, while that for AG was 17.25 mmol/L. ROC curves for ACAG and AG are displayed in Fig. 1, specific parameters of ROC curve analysis are provided in Table 3.

**Table 3:** Comparison of ROC curves for AG, ACAG.

Variable	AUC	95% CI	Optimal cut-off	Sensitivity	Specificity	Youden's index
AG	0.642	0.601–0.683	17.25	0.477	0.765	0.242
ACAG	0.703	0.665–0.741	20.38	0.515	0.819	0.334

### ACAG is associated with 30d risk of death in asthma

**Figure 2:**
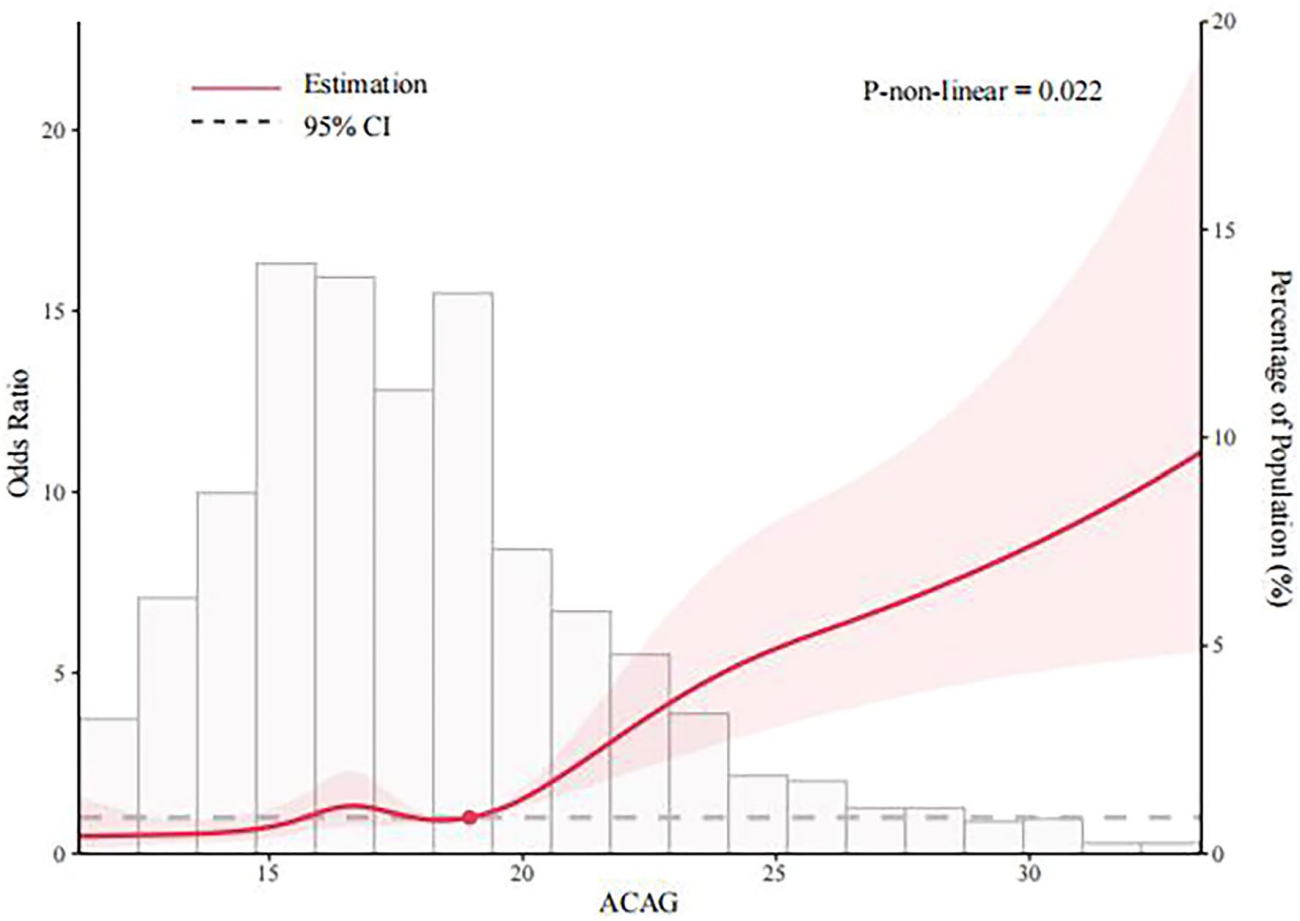
Correlation of ACAG with 30d risk of death in asthma patients.

The results demonstrated that elevated ACAG levels were associated with an increased risk of 30-day mortality in patients with asthma. Specifically, a non-linear relationship was observed between ACAG and mortality (*p* = 0.022)with a cut-off value of 18.95 mmol/L. Notably, when ACAG was below this cut-off value, the OR was close to 1, while patients with ACAG levels above the cut-off value exhibited a significantly higher risk of mortality within 30 days. The RCS plots illustrating this relationship are presented in Fig. 2.

### Comparison of decision curves for AG, ACAG

**Figure 3:**
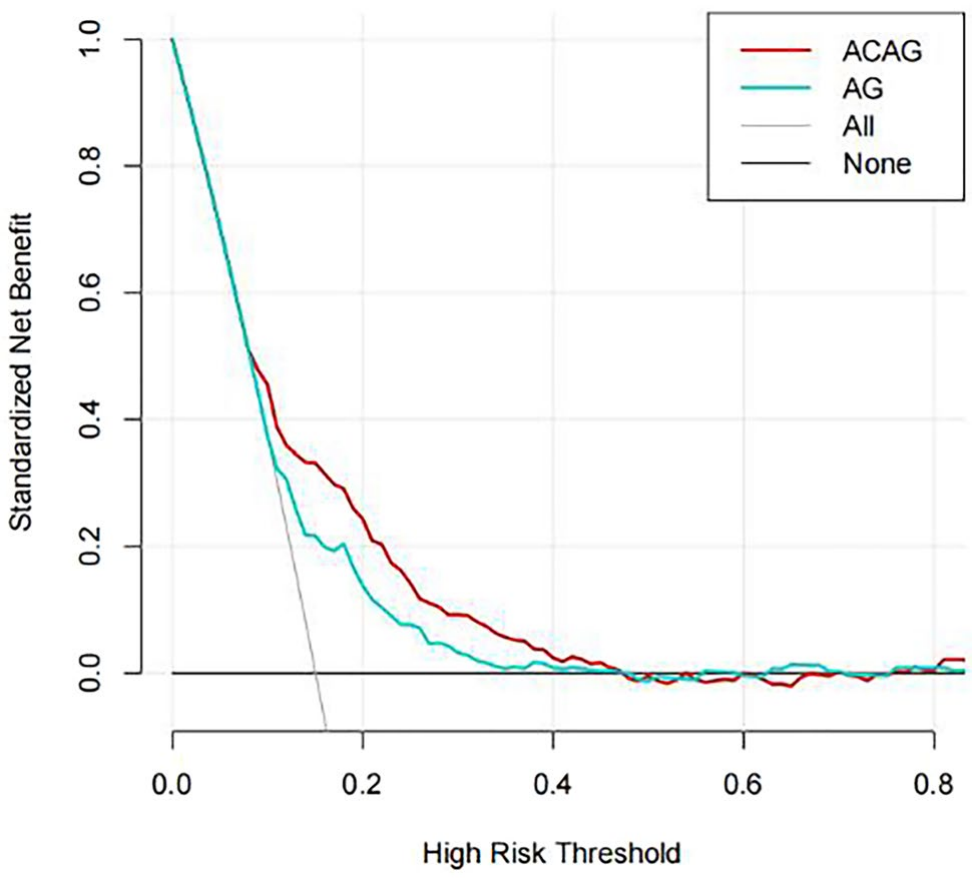
DCA curves for ACAG, AG.

A higher area under the curve (AUC) of the decision curve represents a greater net benefit of the corresponding model. As can be seen from the graph, the net benefit of ACAG is greater than that of AG. The decision curve is plotted in Fig. 3.

### Subgroup analysis

An exploratory subgroup analysis of the relationship between ACAG and 30-d mortality in asthmatic patients was conducted. The subgroups involved included different gender, age, acute phase, and coexisting conditions such as diabetic ketoacidosis and renal insufficiency, which are known to elevate AG levels^16^. The analysis revealed a significant correlation between ACAG and 30-d mortality in all subgroups (*p* < 0.001), and the interaction analysis showed no statistically significant differences among the subgroups *(p* > 0.05), indicating consistency of results across the different groups. The detailed results of the subgroup analysis are shown in Table 4.

**Table 4:** 30d-mortality analysis of ACAG and various subgroups of the population.

Subgroup	Number of patients	OR (95% CI)	*P*-value	*P* for interaction
All parents	1561	1.187 (1.15–1.225)	< 0.001	
Gender				0.542
Male	612 (39.21%)	1.197 (1.139–1.262)	< 0.001	
Female	949 (60.79%)	1.18 (1.134–1.229)	< 0.001	
Age				0.744
≥ 60	762 (48.81%)	1.173 (1.121–1.229)	< 0.001	
< 60	799 (51.19%)	1.206 (1.154–1.263)	< 0.001	
Acute stage				0.975
Yes	1424 (91.22%)	1.187 (1.149–1.227)	< 0.001	
No	137 (8.78%)	1.195 (1.028–1.405)	0.022	
Sepsis				0.355
Yes	710 (45.48%)	1.171 (1.105–1.242)	< 0.001	
No	851 (54.52%)	1.176 (1.132–1.223)	< 0.001	
Renal disease				0.421
Yes	1284 (82.25%)	1.192 (1.15–1.237)	< 0.001	
No	277 (17.75%)	1.16 (1.086–1.245)	< 0.001	
Diabetes				0.33
Yes	1437 (92.06%)	1.191 (1.153–1.231)	< 0.001	
No	124 (7.94%)	1.241 (1.036–1.5)	0.02	

## Discussion

This study highlights the association between ACAG and 30d mortality in patients with asthma in the intensive care unit. Specifically, higher levels of ACAG were found to significantly increase the risk of death. Furthermore, the diagnostic value of ACAG was found to be superior to that of AG, as shown by ROC curve analysis. DCA also demonstrated that ACAG had a greater net clinical benefit than AG. Notably, the subgroup analysis showed that ACAG was associated with 30-day mortality in all subgroups of the population stratified by age, sex, acute exacerbation, comorbid renal disease, and comorbid diabetes.

AG has been widely used in clinical practice to reflect disturbances in the acid–base balance of patients and has been associated with mortality and length of stay in the intensive care unit^17^. It has also been used in previous studies to discuss the prognosis of various diseases, such as cardiogenic shock and sepsis, due to its high value and accessibility^18,19^. However, AG is affected by albumin concentration, and as albumin concentration decreases AG decreases accordingly. In fact, in this study, it was also observed that the albumin concentration in the asthma death group was lower compared to the survival group, possibly due to albumin's role in reflecting the nutritional status and reducing inflammation. And it can also play an anti-inflammatory role in the disease by reducing endothelial cell apoptosis and inhibiting oxidative stress^20^. Thus, for these patients, the direct use of AG values to assess the condition may not be accurate enough.

Due to the limitations of AG as a diagnostic tool and its susceptibility to inaccurate measurements, there has been a growing need for a new index that can provide more accurate predictions of patient outcomes. To address this issue, the concept of ACAG was developed, and in recent years, it has been used to predict the prognosis of various diseases, including coronary artery bypass graft patients and those with acute kidney injury^21,22^. ACAG has been shown to be an independent risk factor for death, and its cut-off values for ACAG vary slightly between diseases, from > 21.25 mmol/L in ICU patients with sepsis to > 20 mmol/L in patients in cardiac arrest^23^. The ROC curve in this study revealed that the cut-off value for ACAG prediction of 30-d mortality in patients with asthma was 20.38 mmol/L, which may be due to differences in the disease spectrum with different albumin concentrations and anion concentration. In contrast, the RCS plot suggested that the patients' risk of death increased significantly when ACAG levels exceeded 18.95 mmol/L. In clinical practice, we can choose a cut-off value of 20 mmol/L to assess the risk of death in patients in order to take into account practicality.

Elevated ACAG levels are common in patients with asthma and may occur due to various reasons. Firstly, hypoxemia or reduced cardiac output can lead to inadequate tissue perfusion and increased lactate production^24^. Secondly, some medications used to treat asthma, such as salbutamol and theophylline, can cause lactic acidosis^25^. Accumulation of other acids in the extracellular fluid, such as beta-hydroxybutyric acid and acetoacetic acid, can also contribute to increased ACAG levels^26^. Hence, ACAG testing is necessary in the asthmatic patients. Another pathophysiological feature of asthma patients admitted to the intensive care unit is their susceptibility to hypo-protein aemia, which can lead to missed detection of lactate and other cryptic anions^27^. In the presence of hypoproteinemia, the detection of lactate and some other cryptic anions can be missed, and it is more appropriate to use ACAG rather than AG to assess the patient's condition^28^. Where direct measurement of AG bias could be as high as 10.2 mmol/L in a group of patients with a 76% prevalence of hypoproteinemia, but the error value was substantially reduced by albumin correction. As some patients in this study had hypoproteinemia, ACAG is more suitable parameter than AG for assessing the condition of this asthmatic population. Our study revealed a nonlinear relationship between ACAG levels and 30-day mortality in patients with asthma. Critically ill patients with asthma often present with hypoproteinemia and are at risk for acid–base disturbances. While ACAG level can make up for AG in albumin levels in patients with abnormal defects, make the body real AG levels. At the same time it inherited the clinical significance of the AG, high level of ACAG patients anoxia serious, produce acid increased, these patients have higher mortality rates. Therefore, ACAG has a good predictive value for 30-day all-cause mortality in patients with asthma.

Our study demonstrated that higher ACAG was associated with an increased risk of 30d-mortality for patients with asthma, emphasizing the importance of clinical attention and intervention to reduce mortality in this population. Our findings suggest that ACAG has better predictive value than traditional indicators AG for mortality in asthma patients, which is consistent with the findings of ACAG in other diseases (e.g. sepsis)^10^. It has good potential to be a clinically useful tool as it reflects tissue perfusion and provides more accurate measurements than directly measured AG values. Additionally, ACAG is accessible and can be used frequently to guide clinical management in the assessment of patients' conditions.

However, there are limitations to our study that should be considered. Firstly, although this study shows that ACAG is associated with the mortality of asthma patients, and has the advantage of easy access and promotion in clinical practice, it also has some shortcomings, such as the lower ROC, sensitivity rate, OR value. We recognize that it is difficult for a single indicator to predict the complex pathophysiological process of asthma death, and in future studies, we may be able to incorporate it into the development of new predictive models to improve its diagnostic effectiveness. Secondly, some patients in the MIMIC IV database did not have their albumin measured, which prevented us from calculating ACAG for those individuals. Thirdly, we only collected ACAG values within 24 h of admission and did not monitor dynamic changes in ACAG levels over time as the patient's condition and treatment may have changed. In addition, many asthma patients in the database are missing indicators of lung function, so it is not possible to study ventilation function tests (including FEV1).Finally, our study was conducted with patients from the US population, and it is uncertain whether our findings are generalizable to other populations. Therefore, further prospective randomized controlled trials are necessary to confirm the results of this study.

## Conclusion

In the present study, we found that higher ACAG scores are associated with an increased risk of mortality in patients with asthma who admitted to the ICU within 30 days of admission. Our results also suggest that ACAG is a reliable predictor of 30-day mortality, exhibiting superior predictive performance compared to AG.

### Supplementary Information

Supplementary Table 1.

## Data Availability

The dataset used in this study can be found on the online website of the MIMIC-IV database. Anyone who meets the requirements for database usage can access the database.
