# Supplementary material for: The relationship between albumin corrected anion gap levels and mortality in patients with asthma in the ICU
Source: Sci Rep. 2023 Oct 6;13:16903. doi: 10.1038/s41598-023-44182-8 (PMC10558512; doi:10.1038/s41598-023-44182-8)
Supplement: Supplementary file 1 — Supplementary Table 1. [file 41598_2023_44182_MOESM1_ESM.pdf]

Supplementary Table1
Definition of asthma

icd_code	long_title	icd_version
J45909	Unspecified asthma, uncomplicated	10
49390	Asthma, unspecified type, unspecified	9
49392	Asthma, unspecified type, with (acute) exacerbation	9
J45998	Other asthma	10
49320	Chronic obstructive asthma, unspecified	9
J45901	Unspecified asthma with (acute) exacerbation	10
J4521	Mild intermittent asthma with (acute) exacerbation	10
49300	Extrinsic asthma, unspecified	9
49322	Chronic obstructive asthma with (acute) exacerbation	9
49391	Asthma, unspecified type, with status asthmaticus	9
J4520	Mild intermittent asthma, uncomplicated	10
J4551	Severe persistent asthma with (acute) exacerbation	10
J4550	Severe persistent asthma, uncomplicated	10
J4541	Moderate persistent asthma with (acute) exacerbation	10
J45902	Unspecified asthma with status asthmaticus	10
J4540	Moderate persistent asthma, uncomplicated	10
J45991	Cough variant asthma	10
J4530	Mild persistent asthma, uncomplicated	10
49302	Extrinsic asthma with (acute) exacerbation	9
49382	Cough variant asthma	9
49301	Extrinsic asthma with status asthmaticus	9
